# Evaluating Temperature Effects on Bluetongue Virus Serotype 10 and 17 Coinfection in *Culicoides sonorensis*

**DOI:** 10.3390/ijms25053063

**Published:** 2024-03-06

**Authors:** Molly Carpenter, Jennifer Kopanke, Justin Lee, Case Rodgers, Kirsten Reed, Tyler J. Sherman, Barbara Graham, Lee W. Cohnstaedt, William C. Wilson, Mark Stenglein, Christie Mayo

**Affiliations:** 1 Department of Microbiology, Immunology and Pathology, Colorado State University, 1601 Campus Delivery, Fort Collins, CO 80526, USA; molly.carpenter@colostate.edu (M.C.); justin.lee@colostate.edu (J.L.); case1prod@gmail.com (C.R.); barb.graham@colostate.edu (B.G.); mark.stenglein@colostate.edu (M.S.); 2 Department of Comparative Medicine, Oregon Health & Science University, Portland, OR 97239, USA; kopanke@ohsu.edu; 3 Wisconsin Veterinary Diagnostic Laboratory, University of Wisconsin-Madison, Madison, WI 53706, USA; kirsten.reed@wvdl.wisc.edu; 4 Diagnostic Medicine Center, Colorado State University, 2450 Gillette Drive, Fort Collins, CO 80526, USA; tyler.sherman@colostate.edu; 5 Foreign Arthropod-Borne Animal Diseases Research Unit, The National Bio and Agro-Defense Facility, USDA Agricultural Research Service, P.O. Box 1807, Manhattan, KS 66505, USA; lee.cohnstaedt@usda.gov (L.W.C.); william.wilson2@usda.gov (W.C.W.)

**Keywords:** bluetongue virus, co-infection, *Culicoides*, next-generation sequencing, reassortment, temperature

## Abstract

Bluetongue virus (BTV) is a segmented, double-stranded RNA virus transmitted by *Culicoides* midges that infects ruminants. As global temperatures increase and geographical ranges of midges expand, there is increased potential for BTV outbreaks from incursions of novel serotypes into endemic regions. However, an understanding of the effect of temperature on reassortment is lacking. The objectives of this study were to compare how temperature affected *Culicoides* survival, virogenesis, and reassortment in *Culicoides sonorensis* coinfected with two BTV serotypes. Midges were fed blood meals containing BTV-10, BTV-17, or BTV serotype 10 and 17 and maintained at 20 °C, 25 °C, or 30 °C. Midge survival was assessed, and pools of midges were collected every other day to evaluate virogenesis of BTV via qRT-PCR. Additional pools of coinfected midges were collected for BTV plaque isolation. The genotypes of plaques were determined using next-generation sequencing. Warmer temperatures impacted traits related to vector competence in offsetting ways: BTV replicated faster in midges at warmer temperatures, but midges did not survive as long. Overall, plaques with BTV-17 genotype dominated, but BTV-10 was detected in some plaques, suggesting parental strain fitness may play a role in reassortment outcomes. Temperature adds an important dimension to host–pathogen interactions with implications for transmission and evolution.

## 1. Introduction

Climate change has been implicated as a determinant in the emergence of diseases caused by arboviruses [1,2,3,4,5]. One such arbovirus is bluetongue virus (BTV), the causative agent of bluetongue disease (BT) that has produced devastating epizootics in domestic and wild ruminant populations. BTV is transmitted to susceptible ruminant species by the *Culicoides* biting midge (Diptera: Ceratopogonidae) with the geographic range of BTV defined by the presence of competent *Culicoides* species [6,7]. Concurrent with climate change, the geographic range of *Culicoides* and BTV is expanding [5,8]. In 2006, BTV serotype 8 (BTV-8) entered Europe and progressed northward causing substantial illness in livestock resulting in losses that were estimated to be over €150 million [9,10,11,12,13]. Increased vector distribution secondary to climate change is commonly postulated as a prominent explanation for these incursions. However, temperature effects on the insect-virus interaction may also have ramifications on BTV evolution and transmission [14,15,16,17].

BTV is the prototype virus of the genus *Orbivirus* (order *Reovirales*, family *Sedaroviridae*) and is non-enveloped with a segmented double-stranded RNA genome [18]. It has ten genomic segments with segment 2 encoding for the viral protein 2 (VP2) that confers the serological response in susceptible hosts. Current BTV nomenclature classifies distinct serotypes corresponding to the host serological response and order of discovery [19,20,21]. There are over 29 recognized BTV serotypes [22]. It important to recognize that while BTV is traditionally classified by serotype, there is additional diversity in the remaining nine genomic segments. As a virus with a segmented genome, reassortment is an important mechanism for BTV evolution [23,24]. Reassortment occurs when two or more parental virus strains exchange genomic segments resulting in progeny with novel segment combinations. Previous studies with BTV and the closely related epizootic hemorrhagic disease virus (EHDV) demonstrated that virogenesis increases in the *Culicoides* vector at warmer temperatures compared to lower temperatures [14,15,25]. Additionally, at higher temperatures, *Culicoides* oogenesis rates accelerate, coupled with earlier oviposition. As the host-biting rate is linked to the rate of oogenesis (due to egg development requiring a bloodmeal), increased temperature may reduce time between blood meals and therefore potentiate virus transmission [26]. However, the impact of temperature on BTV reassortment in *Culicoides* has not been well characterized.

Given the abundance of BTV serotypes, coupled with the possibility of exchange between all ten genomic segments, reassortment has the potential to generate a multitude of novel BTV genotypes. Granted, some requirements for reassortment have been identified that may limit the extent to which different parental BTV strains can naturally reassort. In the field, it is necessary for the parental BTV strains to be co-circulating in the same geographic region (sympatric requirement) and they must be able to infect the same species of host or vector (ecological requirement). Additionally, they must coinfect the same cell (coinfection requirement) and be able to package each other’s genomic segments (segment packaging requirement) within a single virion [27]. The proteins encoded by the parental viruses must be able to function together to productively replicate progeny virus (replicative compatibility requirement) [28]. Finally, after those requirements have been met, progeny reassortants must be able to compete with the parental genotypes (fitness requirement) [29].

Despite the complexity of these requirements, evidence of BTV reassortment is prevalent in experimental and field studies and is predicted to have significant effects on transmission, pathogenicity, and range of host species susceptibility, and therefore requires further investigation [23,30,31,32,33,34,35]. Early studies applied electrophoretic analysis to determine progeny virus genotypes [30,31,33]. However, there are few contemporary studies that have investigated reassortment in *Culicoides* using the highly sensitive next-generation sequencing (NGS) platform. As *Culicoides* are poikilothermic, ambient temperatures of BTV infected *Culicoides* may affect *Culicoides* life traits and virus replication. Therefore, this study aims to compare how ambient temperatures of 20 °C, 25 °C, or 30 °C affect *Culicoides* survival, virogenesis (i.e., viral progeny production), and potential reassortment in *Culicoides sonorensis,* the main vector of BTV in the United States, during coinfection with BTV-10 and BTV-17 [6]. Additionally, we assess virogenesis and *Culicoides* survival during single infections of BTV-10 and BTV-17 to identify if virogenesis of the parental serotype corresponds to progeny virus genotypes and coinfection trends. Understanding consequences of temperature effects on *Culicoides*-BTV interactions (including BTV evolution and *Culicoides*’ survival) will further enhance our understanding of BTV transmission and emergence of novel strains.

## 2. Results

### 2.1. Temperature Affects C. sonorensis Mean Survival

**Figure 1:**
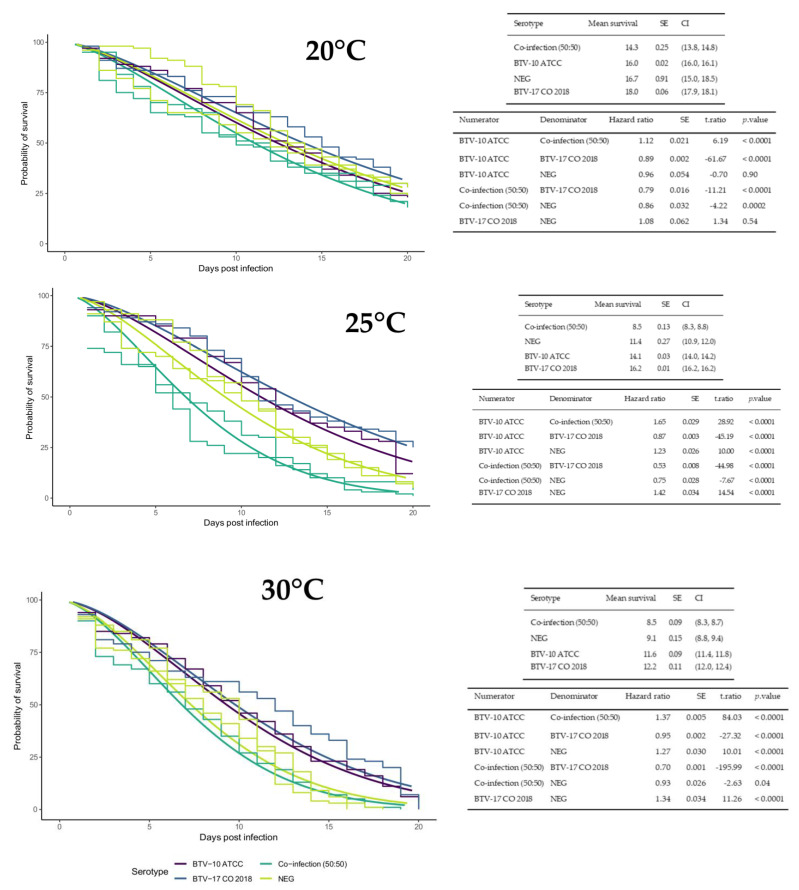
Temperature and BTV infection status impacts *C. sonorensis* lifespan. Plots include all groups from both survival assay trials (experiment 2 and 3) with parametric survival regression models fit to each temperature separately (20 °C, 25 °C or 30 °C) with default Weibull distribution and robust sandwich error calculation. The plots consists of smooth lines for the survival regression curves of each infection group (BTV-10 in purple, BTV-17 in blue, BTV 10:BTV17 coinfected in green, and negative control in yellow) with the *y*-axis representing probability of survival and the *x*-axis representing days post infection. A Kaplan–Meier step curve is included for each group with a separate step curve for the repeated coinfected and negative control groups from experiment 3. Results are shown in associated tables that provide the mean survival days post infection, standard error (SE), and 95% confidence interval (CI) for individual curves and survival odds (Hazard ratio), SE, statistic (t.ratio), and *p*-value for comparisons between curves.

Survival studies were performed for each infection group at each temperature to evaluate if temperature or infection status affected midge lifespan. Across all infection groups, average lifespan decreased as ambient temperature increased (Figure 1). Across all temperature groups, the coinfection group consistently had the lowest mean survival time and BTV-17 had the highest mean survival time. In the 20 °C temperature group, survival differences were not detected between BTV-17 and the negative control, nor BTV-10 and the negative control (Figure 1). In the 25 °C temperature group, there were statistical differences between all infection treatments. Notably, coinfected midges survived on average only about half as long as singly infected by BTV-17 (Figure 1). In the 30 °C temperature group, there were statistical differences among all infection groups except between the coinfected and the negative control group (Figure 1). However, it is important to consider that the hazard ratios reported may be calculated as significantly different because of small standard errors, but might not be different enough for biological meaning (Figure 1).

### 2.2. BTV-10 and BTV-17 Exhibit Different Proportions of Infected Pools of C. sonorensis

**Figure 2:**
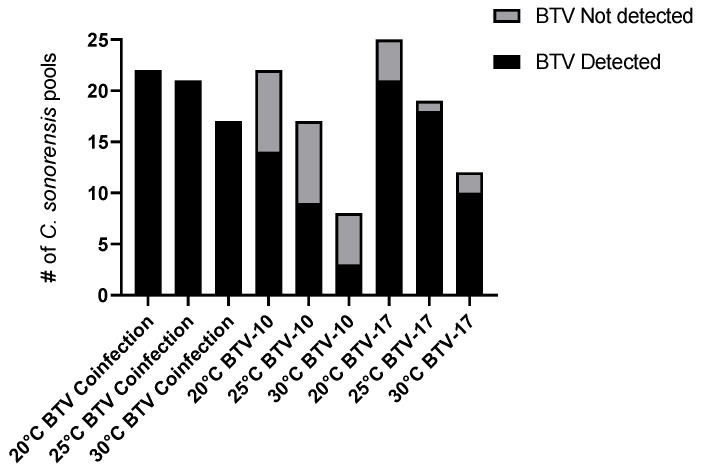
BTV Detected in all pools of the coinfected group. Pool size = 5 *C. sonorensis*. BTV detection was determined by pan BTV qRT-PCR. Black indicates BTV detected and gray indicates BTV not detected.

To determine presence of BTV in midges, pools of five *C.sonorensis* were collected in triplicate from each infection and temperature group every other day and processed for BTV qRT-PCR. BTV was undetectable in several pools of *C. sonorensis* following exposure to infectious bloodmeals (Figure 2). In the 20 °C group, there were 8 of 22 BTV-10 pools (36%) and 4 of 25 BTV-17 pools (16%) that did not have detectable virus. In the 25 °C group, there were 8 of 17 BTV-10 pools (47%) and 1 of 19 BTV-17 pools (5%) that did not have detectable virus. In the 30 °C group, there were 5 of 8 BTV-10 pools (62%) and 2 of 12 BTV-17 pools (17%) that did not have detectable virus. Overall, across all temperatures, BTV-10 exhibited higher proportions of undetectable BTV pools compared to BTV-17, suggesting that *C. sonorensis* may be more competent for BTV-17. Across all temperatures, BTV was detected in all pools of the coinfected groups (Figure 2).

### 2.3. Temperature Affects BTV Virogenesis in C. sonorensis

**Figure 3:**
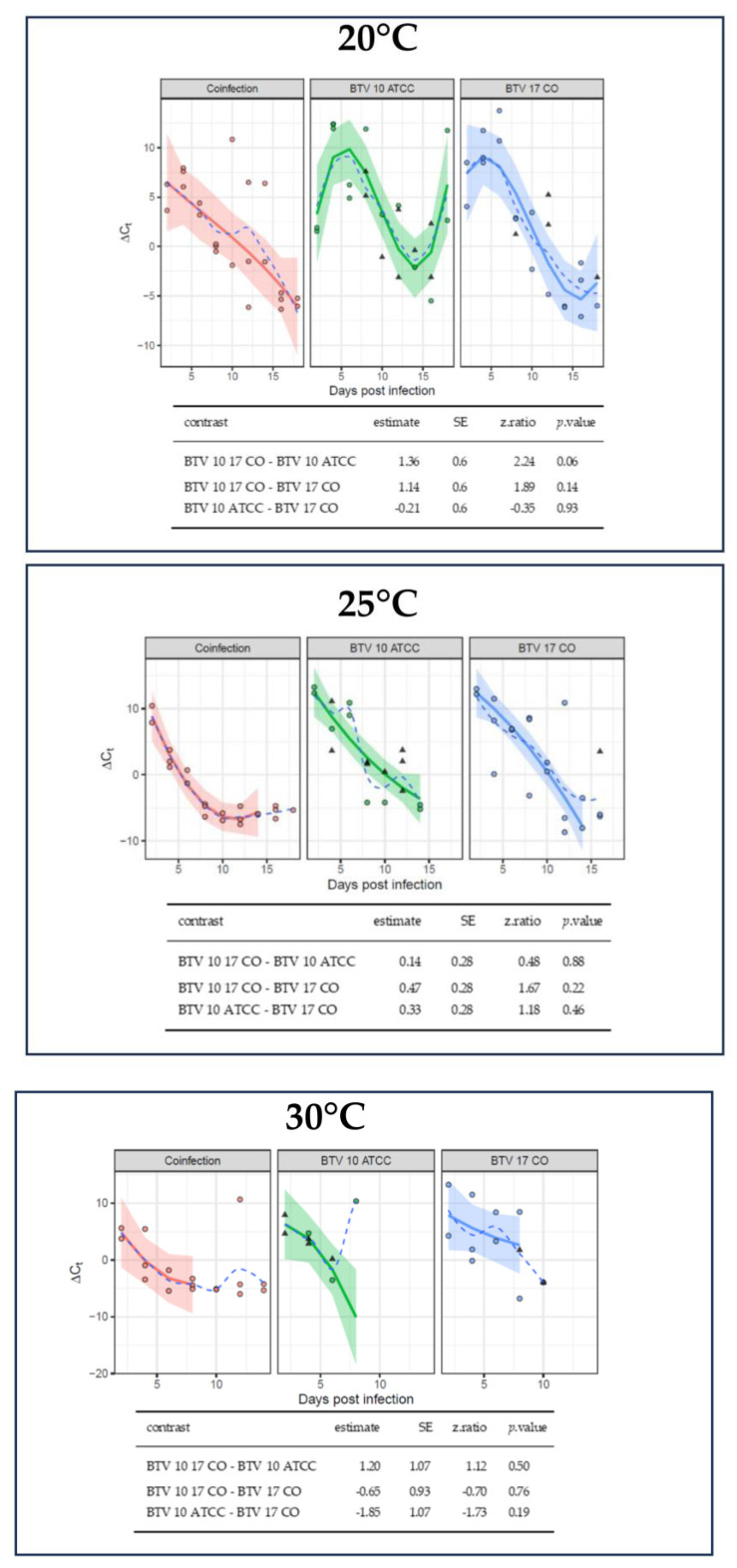
BTV virogenesis is similar across infection groups. Normalization of BTV Ct values calculated for each sample was accomplished using the ∆Ct method based on mean Ct values for BTV and *cox*1 (i.e., Pan BTV Ct−*cox*1 Ct). Due to the decreased survival of *C. sonorensis* at higher temperatures, data collection was limited for the 30 °C collection group. A linear model was fitted to each temperature (20 °C, 25 °C or 30 °C) separately with robust linear models applied to account for outliers. Some pools did not have detectable BTV and are represented by imputed values using multivariate imputation by chained equation with a Bayesian linear regression method and represented by triangles. Circles indicates data points. A third-degree polynomial was fit to Ct values for the 20 °C plot and second-degree polynomials were fit to Ct values for the 25 °C and 30 °C plots. Solid lines indicate the model curve, the shaded region represents the standard error, and the dash line is a curve based on the mean of the data at each day post infection. Pairwise comparisons were performed on the linear portions of the curves with a Tukey HSD adjustment. Estimates of the differences in linear trends, standard error, z-ratio statistic, and *p*-value are given for all comparisons.

Decreased survivability at warmer temperatures impeded comparisons of virogenesis between some temperature groups. Nevertheless, there were notable trends between temperature groups when performing plaque assays (Table 1). At day three post infection, all three pools of *C. sonorensis* reared at 30 °C produced plaques; however, only two of the three pools of *C. sonorensis* reared at 25 °C produced plaques, and none of the three pools of *C. sonorensis* reared at 20 °C produced plaques. *C. sonorensis* pools collected seven days post infection (dpi) and afterwards produced plaques in all temperature treatments. These findings corroborate previous studies and indicate that warmer temperatures correspond with increased early virogenesis. Overall, the decreasing ∆Ct values across infection groups overtime demonstrate that BTV replication was occurring in the midges (Figure 3). The average ∆Ct for the midge pools immediately after imbibing a virus spiked blood meal was −4.9. Midge pools below this ∆Ct suggest that there was amplification of virus [25]. Comparisons of virogenesis between infection groups indicated no significant differences. However, due to several of the BTV-10 pools not being infected, comparison by *p*-values maybe unreliable (Figure 3 and Supplemental Table S1).

### 2.4. BTV-10 and BTV-17 Serotype Detection in Pools of Coinfected C. sonorensis

BTV-17 serotype was overwhelmingly represented in *C. sonorensis* pools from the coinfected group (Table 1). To avoid a previous study issue of sequencing numerous plaques that all aligned to one parental strain, sequencing of pools of midges the plaques were derived from was performed to identify pools that contained both BTV-10 and -17 parental strains [25]. However, due to low reads aligning to BTV-10 or BTV-17, most of the pools could not be adequately characterized. Six pools did have adequate sequencing reads but only demonstrated hits against BTV-17 across all segments, and so plaques from those pools were not sequenced (Table 1). The pools that did not have adequate sequencing reads were evaluated for the presence of segment 2 for both BTV-10 and BTV-17 via serotype-specific qRT-PCR. One pool from 19 dpi at 20 °C and one pool from seven dpi at 30 °C had qRT-PCR detection for both serotypes. There were four pools that had detectable BTV-17 and were inconclusive for BTV-10. Ten plaques were sequenced from pools that had both serotypes represented by qRT-PCR (both with Ct values of 36 and under). Five plaques were sequenced from pools that had both serotypes represented, but one serotype had an inconclusive classification (Ct values of 36 and above). Two plaques were sequenced from pools that only had one serotype detected.

### 2.5. Majority of Plaques from Coinfected C. sonorensis Align with BTV-17

**Figure 4:**
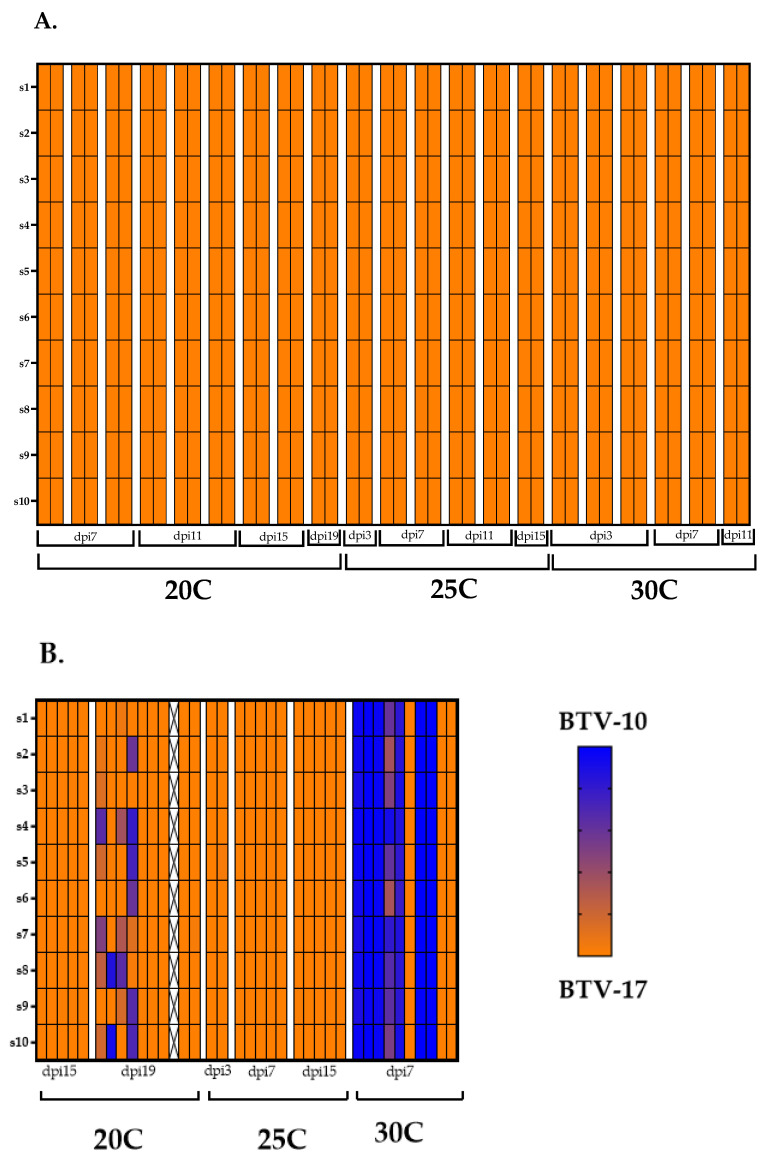
BTV-17 is the predominant plaque genotype. Plaque genotypes that were sequenced from coinfected *C. sonorensis* are depicted by heat maps. (**A**) Represents plaque genotypes from pools that had qRT-PCR detection of only BTV-17 serotype. (**B**) Represents plaque genotypes from pools that had qRT-PCR detection of both BTV-17 and BTV10 serotypes. Each column represents an individual plaque with pools separated by white margins and labeled by dpi. Rows represents the ten BTV segments. Blue indicates that 100% of the sequencing reads for that segment aligned to BTV-10. Orange indicates that 100% of the sequencing reads for that segment aligned to BTV-17. A mixed color gradient indicates that the segment contains reads from both parental strains. X indicates insufficient reads.

**Figure 5:**
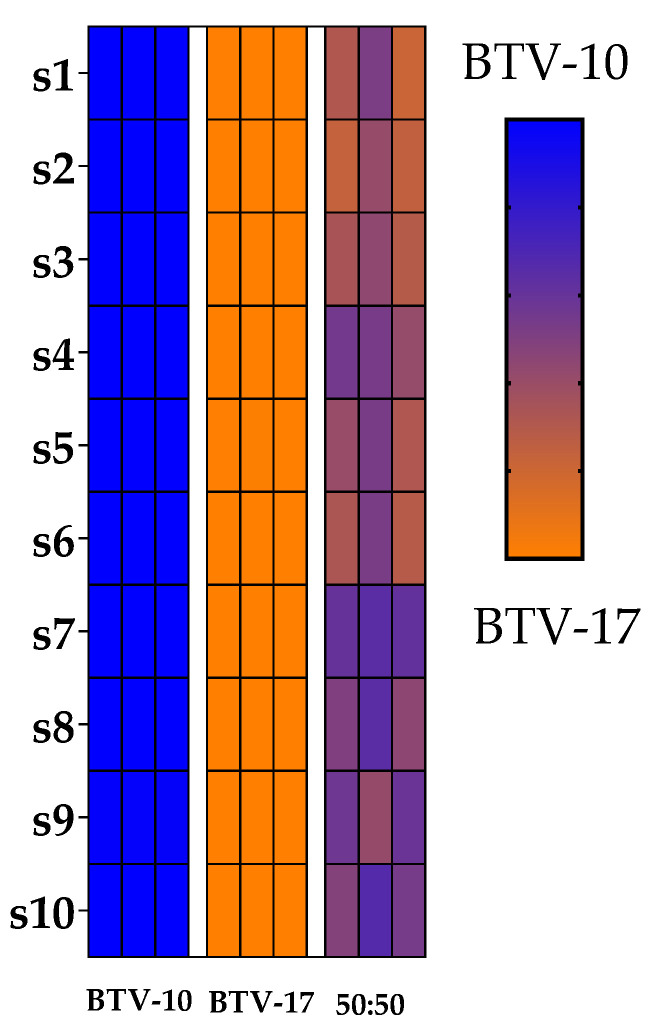
Positive controls of BTV-10, BTV-17, and BTV-10:BTV-17 (at 50:50 ratio) were library prepped and sequenced with genotypes depicted by heatmaps. Rows represents the ten BTV segments. Blue indicates that 100% of the sequencing reads for that segment aligned to BTV-10. Orange indicates that 100% of the sequencing reads for that segment aligned to BTV-17. A mixed color gradient indicates that the segment contains reads from both parental strains.

For genotype assessment, plaques were classified as having either a BTV-10 genotype, a BTV-17 genotype, a reassortant genotype, or a mixed genotype as determined by sequencing reads (Figure 4). As described by Kopanke et al., if >90% of the reads of a segment aligned to one parental strain and all ten segments aligned with the same parental strain, the plaque was classified as having the genotype of that parental strain (BTV-10 or BTV-17). If >90% of the reads of a segment aligned to one parental strain, but not all segments came from the same parental strain, the plaque was classified as a reassortant [36]. Mixed genotypes were derived from plaques that contained at least one segment that aligned to both parental strains (neither parental strain had >90% reads represented in a segment). The 19 dpi pool at 20 °C had four plaques with a mixed genotype. The remainder of the plaques aligned completely with BTV-17 (Figure 4). The seven day post infection pool at 30 °C had four plaques that aligned completely with the BTV-10 parental strain and only three plaques that aligned with the BTV-17 parental strain. The remaining three plaques represented mixed genotypes. None of the plaques were definitively characterized as reassortants. Plaques sequenced from other *C. sonorensis* pools were completely represented by the BTV-17 genotype (Figure 4). Negative water controls, as well as positive controls of BTV-10, BTV-17, and BTV-10:BTV-17 (at 50:50 ratio) indicated that plaque genotypes were appropriately detectable and distinguishable (Figure 5). Overall, these results demonstrate the complexity (and potential stochasticity) of coinfection.

## 3. Discussion

The impetus for this study was the increasing evidence from field and laboratory research that climate change may be accelerating the expansion and emergence of arboviruses such as BTV. To evaluate the consequences of warming temperature effects on *Culidoides*-BTV interactions, we investigated temperature effects on *C. sonorensis* survival, rates of BTV virogenesis, and BTV progeny virus genotype outcomes of *C. sonorensis* coinfected with BTV-10 and BTV-17.

The first component of this study was to evaluate survival of the *C. sonorensis* vector at increasing ambient temperatures. This is an important component of transmission dynamics as the longer an infected vector lives, the more time it has to transmit infection during a subsequent bloodmeal. The survival studies indicated that warmer temperatures correspond with a shorter lifespan. This finding is also consistent with other studies in the literature [25,37,38]. In the context of climate change, the shortened lifespan of a vector may induce a vector competence tradeoff through increased early virogenesis.

In addition to temperature survival trends, observations of infection status and probability of survival were made that highlight the ongoing paradigm shift from arboviruses not affecting the vector toward arboviruses affecting the vector. Midges coinfected with BTV-10 and -17 consistently exhibited shorter average lifespans than single or uninfected midges. Studies from Kopanke et al. observed a similar trend in *C. sonorensis* housed at 20 °C that were exposed via blood meal to BTV-2 and BTV-10, even though this colony demonstrated poor competency for BTV-2 [25]. Virus coinfection studies (specifically Baculoviruses) indicate three different possible outcomes of virus coinfection in the insect vector: synergism (increased mortality), neutralism, and antagonism (decreased mortality) [39,40]. It would be constructive to further investigate if BTV coinfections could overwhelm insect immune defenses and result in decreased lifespans.

In contrast, midges singly infected with BTV-10 and BTV-17 in the 25 °C and 30 °C groups demonstrated increase probability of survival compared to the negative controls. Initially this result seemed counterintuitive. However, the expanding research in the field of insect immunology indicate that outcomes of arbovirus infections can be complex. Chinnaiah et al. found that thrip infection with certain spotted orthotospovirus actually increased vector fitness compared to uninfected control [41]. Insect heat shock proteins activated in response to virus infection are associated with prolonging insect survival [42]. Due to the limitations of our study, including inherent challenges in working with *C. sonorensis* and small standard errors, it is recognized that future studies dedicated to examining BTV infection and *Culicoides* life trait metrics are required.

While virogenesis was difficult to compare between temperature groups due to midge survival differences, plaque assays demonstrated that warmer temperatures yielded earlier production of viral progeny. This finding is corroborated by previous BTV studies as well as in research of other arboviruses [14,25,43,44]. Rates of chemical reactions, which include viral nucleic acid synthesis and viral protein synthesis, increase with temperature [17]. This has been exemplified by measured rates of avian influenza virus hemagglutinin and neuraminidase synthesis and of enzymatic activity of the RNA dependent RNA polymerase at different temperatures [16]. Furthermore, ambient temperature affects insect physiology and immune responses including expression of defensin and cecropin antimicrobial proteins and melanin which may moderate virogenesis [45,46,47].

As virogenesis increases with temperature, we postulated that the frequency of reassortment would also increase with higher temperatures. Due to the small size of the midges and labor considerations, our study utilized pools of midges to perform plaque isolation assays. Moreover, we were reproducing previous research methods in which pools of midges were used because of the wide range of prevalence in which midges obtain disseminated infection [25,48,49]. However, there is a need for further investigation at the individual midge and salivary gland level to add granularity to the characterization of reassortment and transmission. The majority of sequenced plaques isolated from midge pools aligned with the BTV-17 parental genotype regardless of temperature. It is important to iterate that while *C. sonorensis* demonstrated competency for both serotypes, single infection with BTV-17 had more pools of *C. sonorensis* with detectable BTV compared to single infection with BTV-10. Thus, BTV-17 may have higher relative fitness over BTV-10 in this *C. sonorensis* population, which resulted in the overwhelming representation of BTV-17 among the viral progeny. Indeed, there is evidence that vector competence and susceptibility for BTV is under genetic control which can be different between *Culicoides* populations [50]. When developing mathematical modeling of BTV reassortment in midges, Cavany et al. noted that slight differences in the BTV strains may have large effects on reassortment frequency [51]. Similar to our findings, a coinfection study with BTV-1 and BTV-8 in cattle displayed predominant detection of BTV-8 even though cattle demonstrated equivalent susceptibility to both serotypes during single infections [52,53]. Explanations for this finding included biased detection of BTV-8 segment 2 via RT-qPCR (as sequencing was not performed in this study), viral interference, or increased BTV-8 tropism in the examined tissues.

The particular BTV strains employed for the coinfections may also affect reassortment frequency. Successful reassortment requires that the parental strains coinfect the same cell, package with each other’s genomic segments, and have replicative compatibility. Superinfection exclusion, in which a viral infection already occupying a cell prevents a second infection from another virus, is a mechanism that inhibits cellular coinfection. The literature suggests that superinfection exclusion is less effective between viruses that are not as closely related [54]. Thus, requirements for segment packaging and replicative compatibility may favor reassortment between viruses that are more closely related [55]. It remains unclear whether genomic differences in parental strains may facilitate or hinder reassortment potential.

One pool of *C. sonorensis* at seven dpi at 30 °C and one pool of *C. sonorensis* at 19 dpi at 20 °C exhibited progeny virus with mixed genotypes (detection of both parental strains within one segment). A second round of plaque purification was not performed to confirm the identity of mixed plaques, due to concerns of imposing too much plaque bias. Previous research has excluded mixed plaques from the results [25,31,33,36]. However, the data were included in this study for the sake of transparency, particularly in recognition of the potential reasons for mixed plaques. The detection of mixed genotypes could be an artifact of plaque bleed over, in which two plaques may be close together and appear as one plaque, or single plaques formed from aggregated virions [56]. Moreover, BTV has demonstrated egress from cells in lysosome-derived extracellular vesicles [57]. If extracellular vesicles contain both BTV-10 and BTV-17 progeny virus and infect a single cell, this could confound the plaque isolation approach for identifying reassortment. Similarly, in Zika virus, it has been demonstrated that infectious units that form plaques are often a result of several genomes [58]. Hypothetically, occurrences of anomalous segment packing could result in mixed plaques; however, current studies have indicated that BTV undergoes selective segment packaging [27,59,60]. As mixed plaques were found in earlier time points in the 30 °C group as compared to the 20 °C group, it would be compelling to investigate if reassortment frequencies could also be affected by duration of coinfection. Plaquing individual midges and salivary glands in addition to sequencing of more plaques across all temperature groups over time is an approach that will more thoroughly interrogate BTV reassortment.

The overwhelming outcome of parental genotype representation in progeny plaques also highlights the delicate equilibrium between genetic stability and diversity for maintaining fitness in an alternating host cycle while also having mechanisms for genetic alterations for adaption in changing environments. As a parental serotype has demonstrated its capacity to survive the gauntlet of the alternating host cycle, any genetic changes may threaten its ability to replicate when transmitted to the next host. However, this must be balanced with evolution, where there is the promise of increased fitness. Thus, the host–pathogen interaction is the fulcrum for potential viral emergence. In the context of climate change, these mechanisms require further interrogation, particularly in viruses of high consequence such as BTV.

## 4. Materials and Methods

### 4.1. Viruses, Virus Titration, and Cell Culture

The BTV strains employed in this study were selected because they are endemic to the United States and contain sufficient genomic divergence for differentiation by sequencing (Table 2). BTV-10 California 1952 (BTV-10) (Bluetongue virus, type 10, strain 8, ATCC® VR-187™) was procured from ATCC and passaged eight times on BHK 21 cells [61]. BTV-17 Colorado 2018 (BTV-17) was isolated on CuVaW3 cells from BTV-positive whole blood and passaged one time on BHK 21 cells [62]. As both serotypes were isolated in the United States, it was anticipated that the colony *C. sonorensis* would exhibit competence for both serotypes. Whole-genome sequences of each virus were performed previously by our lab and are available on GenBank (BTV-10 GenBank Accession MW456747-MW456756; BTV-17 GenBank Accession OQ798198-OQ798207) [36,62].

Infectious titers for BTV-10 and BTV-17 were determined by 50% tissue culture infectious dose (TCID_50_) in triplicate with negative controls and calculated using the Reed-Muench method [63]. In brief, eight 10-fold dilutions were prepared for each virus in Eagle’s Minimum Essential Medium with Earle’s salts and without L-glutamine (EMEM) (Corning Cell-Gro). 50 µL of each virus dilution was added sequentially toa 96-well plate containing 50 µL EMEM for sample wells. No virus dilutions were added to C = control wells which contained 100 µL of EMEM. Approximately 1.55 × 10^4^ BHK 21 cells were added to each well. The inoculated plates were incubated at 37 °C with 5% CO_2_ for 96 h. After incubation, crystal violet solution was introduced into the wells for staining of viable cells [36].

BHK 21 cells were propagated in BHK 21 media comprising of EMEM, 10% insect cell culture tested fetal bovine serum (certified, heat inactivated, US origin from Sigma Aldrich, St. Louis, MO, USA), 10% tryptose phosphate broth (Sigma Aldrich), and 1% penicillin streptomycin (10,000 U/mL). The cells were incubated at 37 °C with 5% CO_2_ and passaged every three to four days when culture flasks were 80–90% confluent [36].

### 4.2. C. sonorensis Infection and Maintenance

*C. sonorensis* were supplied by the Arthropod-Borne Animal Diseases Research Unit (United States Department of Agriculture, Agricultural Research service, Manhattan, KS, USA), specifically from the AK colony that originated from the field in Owyhee Co., Idaho, August 1973 [64]. *C. sonorensis* were shipped overnight by air at one day of age and held at 25 °C, with a 12:12 h light cycle, and 10% (*w*/*v*) sugar water ad libitum for two days before infection studies commenced.

Defibrinated sheep blood (Hemostat Laboratories, Dixon, CA, USA) was screened for BTV virus and antibody by pan BTV qRT-PCR and the Bluetongue cELISA kit (VMRD, Pullman, WA, USA), respectively. Single infection blood meals were prepared by spiking defibrinated sheep blood with respective BTV serotypes to produce a viral titer of 1.0 × 10^5^ meanTCID_50_/mL. Blood meals containing both serotypes were prepared by spiking defibrinated sheep blood to produce equal titers of 5.0 × 10^4^ TCID_50_/mL of BTV-10 and BTV-17 for a total viral titer of 1.0 × 10^5^ TCID_50_/mL. Infection groups included single infections of BTV-10, single infections of BTV-17, coinfections of BTV-10 and BTV-17, and uninfected blood for negative controls. *C. sonorensis* groups were fed blood meal treatments for a duration of one hour and 30 min via glass bell jar feeders with parafilm membranes and water circulated through a 37 °C water bath to warm the blood [25,65].

After receiving the bloodmeal, *C. sonorensis* groups were chilled for four minutes at −20 °C and placed on a modified chill table to facilitate sorting and collection of engorged females from each infection group into containers to be housed at 20 °C, 25 °C, or 30 °C (Table 3). In total, nine treatment groups were created to assess BTV-10 and -17 single infection and coinfection dynamics under three temperature conditions (Table 3). Additionally, uninfected negative controls were maintained at 20 °C, 25 °C or 30 °C for survival studies and to ensure the absence of BTV contamination through the experimental period. Immediately after feeding, 5–10 blood-fed females from each infection group were collected to assess uptake of virus via pan BTV qRT-PCR. For the duration of the experimental period, *C. sonorensis* treatment groups were maintained in separate incubators with a 12:12 h light cycle and housed in containers of non-treated paper (Rigid Paper Tube Corporation, Wayne, NJ, USA) topped with sheer pantyhose over the lid for access to feeding and air exchange. Sugar water prepared at 10% (*w*/*v*) was provided ad libitum via cotton wool and uninfected blood meals were provided every four days for 30 min. Humidity and temperatures of incubators were monitored daily.

Due to constraints in the number of *C. sonorensis* individuals that could be procured and housed at the same time, different experiments of this study were performed at separate times. First, the *C. sonorensis* BTV-10 and BTV-17 co-infection experiment was performed (experiment 1). Next, BTV-10 and -17 single infections, in addition to survival studies, were performed (experiment 2). Finally, an additional survival study was performed to expand upon data collected in experiment 2 (experiment 3). The parameters for the experiments are outlined in Table 3 for the qRT-PCR and plaque assays and Table 4 for the survival assays. The same BTV stocks were employed for each experiment and negative controls were included for each experiment in this study.

### 4.3. C. sonorensis Survival Studies

*C. sonorensis* survival studies were performed for each temperature and infection group combination including negative controls. *C. sonorensis* from each representative infection group were housed 50 midges per container in duplicate. Daily survival counts were performed and recorded (Table 4).

### 4.4. C. sonorensis Collections

To longitudinally evaluate virogenesis via pan BTV qRT-PCR, five *C. sonorensis* were collected in triplicate from each infection group at each temperature every other day. For the production of plaque assays to assess progeny genotypes, pools of ten *C. sonorensis* from the coinfection groups at each temperature were collected in triplicate on days 3, 7, 11, 15, and 19 [25]. *C. sonorensis* were collected from each group until none remained.

### 4.5. Coinfection Plaque Assays

The *C. sonorensis* pools collected for plaque assays were immediately processed for plaque isolation. First, 500 µL of EMEM was added to each pool of ten *C. sonorensis* and then manually homogenized with a sterile pestle, vortexed, and centrifuged. The supernatant was sterile-filtered with a syringe filter (0.22 µM millex-GV syringe filter, MilliporeSigma, Burlington, MA, USA). Fifty µL of the supernatant was reserved for nucleic acid extraction and downstream serotype-specific qRT-PCR and sequencing. The remaining supernatant was used to make dilutions of 1:2, 1:10, 1:100, 1:1000, and 1:10,000 in EMEM for plaque assays. Six-well plates with a confluent monolayer of BHK 21 cells were used for the plaque assays. Confluency was achieved by seeding BHK 21 cells (1.0 × 10^5^ cells/well) with BHK 21 media, as described above, and incubated at 37 °C with 5% CO_2_ for 48 h prior to the plaque assays. Immediately before inoculation with *C. sonorensis* supernatant dilutions, each well was washed with 0.5 mL PBS (pH 7.4). Five hundred µL of each *C. sonorensis* supernatant dilution was applied into each well, including a negative control well that contained media only. The inoculated 6-well plates were placed in 37 °C with 5% CO_2_ incubators for one hour and gently rocked every 15 min to facilitate even dispersion of the virus. The inoculum was gently aspirated off the cell monolayers and each well washed with 0.5 mL PBS (pH 7.4). Two mL of an overlay mixture containing 3:1 BHK 21 media to 2% agarose in Earle’s Buffered Salt Solution (EBSS) warmed to 40 °C was applied to each well. The 6-well plates were incubated at 37 °C with 5% CO_2_ and observed twice daily for visualization of plaques. When plaques first appeared (approximately 48 h after incubation) a second overlay of 1 mL/well of 3:1 BHK 21 media to 2% agarose in EBSS with 0.1% neutral red stain was added and the plates were incubated for a further 8–24 h at 37 °C until visible plaques were apparent.

For plaque propagation, individual plaques from the 6-well plates were harvested using a 1000 µL barrier filter pipette tip and each plaque was placed in an individual well of the 48-well plate that contained BHK 21 cells (4.65 × 10^4^ cells/well). The 48-well plates with the harvested plaques were incubated at 37 °C with 5% CO_2_ and observed twice daily for cytopathic effects (which usually occurred after three to four days). When 3 to 4 + cytopathic effect was observed in a well, the propagated virus isolate was collected and stored at −80 °C until extraction and library preparation for NGS.

### 4.6. Nucleic Acid Extraction and DNase Treatment

All pools of *C. sonorensis* (n = 5) collected in triplicate on days 4, 8, 12, and 16 post infection were processed for qRT-PCR. Two of the three pools (n = 5) were processed from *C. sonorensis* collected on days 2, 6, 10, 14, and 18 dpi. Some pools were reserved for potential individual analysis. For processing, each pool was manually homogenized with a sterile pestle in 250 µL EMEM, vortexed, and centrifuged. Fifty µL of the supernatant was transferred to a 96-well plate for nucleic acid extraction. Nucleic acid extraction was performed using the MagMAX Pathogen RNA/DNA Kit (Applied Biosystems, Foster City, CA, USA) according to the manufacturer’s low-cell content protocol using a KingFisher Flex robot (Thermo Fisher, Waltham, MA, USA) with a 90 µL elution volume. After extraction, samples were treated with DNase 1 (Thermo Fisher). Briefly, 2 µL of DNase 1 and 2 µL of 10× reaction buffer were added to 12 µL of extracted sample and incubated at 37 °C for 30 min. To inactivate the DNase, 2 µL of EDTA was then added to each sample and incubated at 65 °C for 10 min [25].

The propagated plaques and the supernatants from the homogenized *C. sonorensis* pools that were used to isolate the plaques were extracted using the same kit, protocol, and input volume as above; however, they were processed manually with a 45 µL final elution volume. The extracts were then treated with the TURBO DNA-*free*™ kit (Invitrogen, Vilnius, Lithuania). In brief, the TURBO DNA-*free*™ treatment protocol used 2–4 U of DNase in 0.1 volume of DNase buffer per sample and incubates for 30 min at 37 °C. Inactivation of DNase, 8 µL of inactivation reagent were added with 5 min of incubation at room temperature. The samples were centrifuged (10,000× *g* for 1.5 min) and supernatant collected. The supernatant was treated with LiCl with a 2.0 M final concentration for selective precipitation of single-stranded RNA. The samples were incubated for 16 h at 4 °C during precipitation and then centrifuged (18,000× *g* at 4 °C for 20 min). Supernatant was collected and remaining salt was removed using a 1.25× MagMAX Pathogen RNA/DNA kit clean-up protocol per manufacturer’s recommendations [25].

### 4.7. Pan BTV and cox1 qRT-PCR Assay

Each extracted, DNase-treated *C. sonorensis* pool was processed in duplicate for the detection of BTV using “pan-BTV” qRT-PCR primers as described by Kopanke et al. [25,66]. Additionally, qRT-PCR of the *Culicoides* mitochondrial cytochrome *c* oxidase subunit 1 (*cox*1) gene was performed on each sample in duplicate to normalize for extraction efficiency. A FAM-based probe (3′FAM-TGAATACTT/ZEN/CCTCCTTCTCTTTCTT-3IABkFQ/5′) was designed previously by our lab based on available GenBank sequences of this gene [25,66,67,68,69]. Analysis of *cox*1 expression was also performed as described by Kopanke et al. [66]. Positive and negative controls for both BTV and *Culicoides cox*1 were run with each PCR plate, as well as a reverse transcriptase-free (no-RT) control was run to ensure that DNAse treatment was effective (i.e., no detection of *cox*1 in absence of reverse transcriptase). A Ct value less than 40 was considered positive for detection. Normalization of BTV Ct values were calculated for each sample using the ∆Ct method based on mean Ct values for BTV and *cox*1 (i.e., Pan BTV Ct—*cox*1 Ct) [25].

To determine if both BTV-10 and BTV-17 were present in pools of *C. sonorensis* used for plaque propagation, a BTV-10 and BTV-17 serotype-specific qRT-PCR was performed as described by Maan et al. [70]. A Ct value less than 36 was considered positive for detection.

### 4.8. Library Preparation and Whole-Genome Sequencing

To assess the genotype of each of the ten BTV segments for each propagated plaque, shotgun whole-genome sequencing was performed on the extracted, DNAse-treated, and LiCl-treated samples. With each library preparation and sequencing run, negative water controls, as well as positive controls of BTV-10, BTV-17, and BTV-10:BTV-17 (at 50:50 ratio) were included. The NEBNext^®^ Ultra II Directional RNA Library Prep Kit of for Illumina^®^ (New England BioLabs Inc., Ipswich, MA, USA) was used to prepare sample libraries. Section 4 Protocols (for use with Purified mRNA or rRNA depleted RNA) were performed with the following modifications for double-stranded RNA: (1) RNA fragmentation at 90 °C for one minute, and (2) first strand cDNA synthesis reaction was processed for ten minutes at 25 °C, 30 min at 42 °C, and 15 min at 70 °C. For sample identification, NEBNext Multiplex Oligos for Illumina (96 Index Primers) were used according to the manufacturer’s instructions. Libraries were assessed for dsDNA quality and concentration using a Qubit 2.0 fluorometer (Thermo Fisher, Waltham, MA, USA) and High Sensitivity D1000 DNA screentapes (Agilent, Santa Clara, CA, USA) on the TapeStation 2200 or 4150 instruments (Agilent, Santa Clara, CA, USA).

Pools of approximately 60 samples were processed at a time and subsequently size-selected for inserts between 300 and 900 base pairs with base pair size selection on a 1.5% agarose gel. The desired region of the gel was excised and was reextracted using the QIAquick Gel Extraction kit according to the manufacturer’s protocol (Qiagen, Hilden, Germany). A final bead-clean up following Section 4.9 of the NEBNext^®^ Ultra II Directional RNA Library Prep kit for Illumina^®^ was performed to optimize nucleic acid concentrations. After, the pool was analyzed using a Qubit fluorometer and TapeStation instruments as described above. Libraries were quantified using the KAPA Library Quantification kit (KAPA Biosystems, Basel, Switzerland) according to manufacturer’s protocol. To perform paired-end sequencing (2 × 150), the NextSeq 500 instrument (Illumina, San Diego, CA, USA) was used with the NextSeq 500/550 Mid Output kit v2.5 (300 cycles).

### 4.9. BTV Analysis Pipeline and Bioinformatics

Libraries were demultiplexed and analyzed with the stenglein-lab/btv_segment_table pipeline “https://github.com/stenglein-lab/btv_segment_table (accessed on 18 May 2023)”. This pipeline uses modules and infrastructure developed by Nextflow and the nf-core community [71,72]. The objective of this pipeline is to quantify the number of parental virus reads that map to each of the ten BTV genomic segments to facilitate assessment of genotype and reassortment events. In brief, Cutadapt performed trimmings of low-quality bases and adapter sequences [73]. Cd-hit was used to collapse duplicate read pairs, while Bowtie2 performed read alignments to the reference sequences (BTV-10 and BTV-17) that we provided [74,75,76]. The pipeline was run with default parameters with the exception of the BTV Reference Sequences. FASTA files of BTV-10 and BTV-17 reference sequences (BTV-10 GenBank Accession MW456747-MW456756; BTV-17 GenBank Accession OQ798198-OQ798207) used in this study were formatted and executed with the --refseq_fasta command line option.

### 4.10. Statistical Analysis

For survival analysis, parametric survival regression models were fit to each temperature plot separately with a default Weibull distribution and robust sandwich error calculation [77,78]. Additionally, a Kaplan–Meier step curve was included. Mean survival times were calculated by survival regression with standard error and 95% confidence intervals. Comparisons between infection groups of survival odds is expressed as the ratio. Standard error of the ratio and *p*-value were also determined. Shorter survival times of *C. sonorensis* at warmer temperatures impeded comparisons of virogensis between temperature groups. Additionally, BTV-10 had several pools of *C. sonorensis* were BTV could not be detected. To accommodate for this, absent Ct values were imputed using multivariate imputation by chained equation with a Bayesian linear regression method [79,80,81]. To account for outliers, robust linear methods were employed [82]. For the 20 °C temperature groups, a third-degree polynomial was fit to the Ct values. For both the 25 °C and 30 °C temperature groups, a second-degree polynomial was fit to the Ct value. Pairwise comparisons of the linear portions between infection groups within a temperature treatment were made with a Tukey HSD adjustment. Estimates of differences in linear trends, standard error of the difference, z-ratio statistic and *p*-values were determined. Statistics for qRT-PCR and survival analysis were conducted in open source software R version 4.2.1. Bar graphs and heat maps of plaque genotypes were produced using GraphPad Prism 8.1.0 (321).

## Figures and Tables

**Table 1:** Detection of viable BTV and serotyping of *C. sonorensis* pools used for plaque assays. Tables indicate which pools were able to generate viable BTV for plaque isolation assays. Additionally, *C. sonorensis* pools were screened for BTV-10 and BTV-17 via serotype-specific qRT-PCR. Presence of viable virus and serotype detection are indicated by (+). Absence of viable virus and serotype not detected is indicated by (−). (I) indicates an inconclusive serotype result (Ct > 36). Days where *C. sonorensis* were not available for plaque assay are indicated by (n/a). Additionally, pools of *C. sonorensis* that did not produce viable virus were not screened for BTV-10 or BTV-17 and are indicated by (−). Finally, plaques from pools that had adequate sequences reads aligning to our BTV-17 reference sequence were not processed for further sequencing and are indicated by (0) in the No. Plaques Sequenced column.

			20 °C				25 °C				30 °C		
			PCR Serotype Detection				PCR Serotype Detection				PCR Serotype Detection		
dpi	Pool	Plaque Assay	BTV-10	BTV-17	No. Plaques Sequenced	Plaque Assay	BTV-10	BTV-17	No. Plaques Sequenced	Plaque Assay	BTV-10	BTV-17	No. Plaques Sequenced
	A	−	n/a	n/a	n/a	+	I	+	2	+	−	+	2
3	B	−	n/a	n/a	n/a	+	−	+	2	+	−	+	2
	C	−	n/a	n/a	n/a	−	n/a	n/a	n/a	+	−	+	2
	A	+	−	+	2	+	I	+	5	+	+	+	10
7	B	+	−	+	2	+	−	+	2	+	−	+	2
	C	+	−	+	2	+	−	+	2	+	−	+	2
	A	+	−	+	2	+	−	−	2	+	−	+	2
11	B	+	−	−	2	+	−	−	2	+	−	+	0
	C	+	−	+	2	+	−	+	0	+	−	+	0
	A	+	I	+	5	+	I	+	5	+	−	+	0
15	B	+	−	+	2	+	−	+	0	n/a	n/a	n/a	n/a
	C	+	−	+	2	+	−	+	2	n/a	n/a	n/a	n/a
	A	+	−	+	2	n/a	n/a	n/a	n/a	n/a	n/a	n/a	n/a
19	B	+	−	+	0	n/a	n/a	n/a	n/a	n/a	n/a	n/a	n/a
	C	+	+	+	10	n/a	n/a	n/a	n/a	n/a	n/a	n/a	n/a

**Table 2:** Nucleotide pairwise identity of BTV-10 and BTV-17 genomic segments.

Segment	Protein Encoded	Percent Pairwise Identity
Seg-1	VP1	96.1
Seg-2	VP2	68.8
Seg-3	VP3	97.3
Seg-4	VP4	96.3
Seg-5	NS1	96.9
Seg-6	VP5	79.5
Seg-7	VP7	95.8
Seg-8	NS2	96.2
Seg-9	VP6/NS4	96.9
Seg-10	NS3/NS3A/NS5	81.7

**Table 3:** *C. sonorensis* BTV infections for PCR and plaque assays in experiments 1 and 2.

		BTV-10			BTV-17			BTV-10 and 17		Negative	
Temperature	20 °C	25 °C	30 °C	20 °C	25 °C	30 °C	20 °C	25 °C	30 °C	25 °C	25 °C
Experiment ID	2	2	2	2	2	2	1	1	1	1	2
Number of Midges per	n = 150	n = 151	n = 150	n = 151	n = 153	n = 156	n = 400	n = 407	n = 407	n = 103	n = 150
container	n = 150	n = 194	n = 128	n = 194	n = 183	n = 191	n = 425	n = 247	n = 339		n = 69
Mean Bloodmeal BTV Titer (TCID_50_/mL)	1.0 × 10^5^	1.0 × 10^5^	1.0 × 10^5^	1.0 × 10^5^	1.0 × 10^5^	1.0 × 10^5^	BTV-10: 5.0 × 10^4^ BTV-17: 5.0 × 10^4^	BTV-10: 5.0 × 10^4^ BTV-17: 5.0 × 10^4^	BTV-10: 5.0 × 10^4^ BTV-17: 5.0 × 10^4^	-	-

**Table 4:** *C. sonorensis* BTV infections for survival assays in experiments 2 and 3.

		BTV-10			BTV-17			BTV-10 and 17			Negative	
Temperature	20 °C	25 °C	30 °C	20 °C	25 °C	30 °C	20 °C	25 °C	30 °C	20 °C	25 °C	30 °C
Experiment ID	2	2	2	2	2	2	2 and 3	3	3	2 and 3	2 and 3	2 and 3
Survival groups	n = 50 in duplicate	n = 50 in duplicate	n = 50 in duplicate	n = 50 in duplicate	n = 50 in duplicate	n = 50 in duplicate	n = 50 in duplicate	n = 50 in duplicate	n = 50 in duplicate	n = 50 in duplicate	n = 50 in duplicate	n = 50 in duplicate
Mean Bloodmeal BTV Titer (TCID_50_/mL)	1.0 × 10^5^	1.0 × 10^5^	1.0 × 10^5^	1.0 × 10^5^	1.0 × 10^5^	1.0 × 10^5^	BTV-10: 5.0 × 10^4^ BTV-17: 5.0 × 10^4^	BTV-10: 5.0 × 10^4^ BTV-17: 5.0 × 10^4^	BTV-10: 5.0 × 10^4^ BTV-17: 5.0 × 10^4^	-	-	-

## Data Availability

Data are contained within this article or Supplementary Materials.

## Supplementary Materials

The following supporting information can be downloaded at: https://www.mdpi.com/article/10.3390/ijms25053063/s1.
