# Supplementary material for: Evaluating Temperature Effects on Bluetongue Virus Serotype 10 and 17 Coinfection in Culicoides sonorensis
Source: Int J Mol Sci. 2024 Mar 6;25(5):3063. doi: 10.3390/ijms25053063 (PMC10932384; doi:10.3390/ijms25053063)
Supplement: Supplementary file 1 [file ijms-25-03063-s001.zip › ijms-2776885-supplementary.pdf]

Supplementary Table S1. Pan BTV qRT-PCR ct values in duplicate for infected *C. sonorensis* pools at temperatures 20°C, 25°C, and 30°C. UD indicates an undetermined ct. N/A indictates no midges were alive for sampling.

20C Temperature		Pool A		Pool B		Pool C	
**Infection Group**	**DPI**	**ct1**	**ct2**	**ct1**	**ct2**	**ct1**	**ct2**
**BTV-10 & 17**	2	29.1	28.9	32.4	33.41		
	4	32.4	32.3	33.7	32.9	31.9	31.1
	6	29.4	29.5	28.9	29		
	8	25.2	25.3	29.1	29	26.7	27.4
	10	36.8	36.8	24.5	24.1		
	12	33.4	33.28	24.4	24.5	21	20.5
	14	23.9	24	32.7	32.8		
	16	20	20.1	21.8	23.2	21.8	21.8
	18	20.4	21.8	21	21.1		
**BTV-10**	2	29.5	29.4	28.8	28.6		
	4	38.4	37.7	39.6	37.7	38.3	39.7
	6	33.4	33.5	33.2	32.6		
	8	UD	UD	UD	UD	36.7	39.5
	10	UD	UD	31.7	29.9		
	12	30.8	30.1	UD	UD	UD	UD
	14	UD	UD	24.7	24.4		
	16	UD	UD	UD	UD	22.9	22.9
	18	30.3	30.2	39.1	39.8		
**BTV-17**	2	29.4	29.8	32.7	33.1		
	4	35.9	36.1	39.2	37.5	34.9	35.1
	6	38.8	38.7	35.6	36.2		
	8	29.6	29.5	UD	UD	29.5	29.2
	10	30.4	30.3	24.2	24.9		
	12	UD	UD	22.5	22.5	UD	UD
	14	23.2	23.2	24.5	23.8		
	16	25	25	23.3	23.7	20.5	20.5
	18	23.7	21.2	UD	UD		
**25C Temperature**		**Pool A**		**Pool B**		**Pool C**	
**Infection Group**	**DPI**	**ct1**	**ct2**	**ct1**	**ct2**	**ct1**	**ct2**
**BTV-10 & 17**	2	32.3	32.4	34.5	34.9		
	4	26.9	27.1	29.7	29.5	27.6	27.2
	6	26.3	26.7	23.4	25.5		

	8	22.2	22	21.1	21.1	20.2	20.3
	10	19.4	19.4	19.7	19.6		
	12	19.6	19.2	19.2	19	21.6	21.6
	14	20.2	20.3	20.3	20.2		
	16	21.5	21.5	24.7	25.4	23.9	21.1
	18	23.3	23	N/A	N/A		
**BTV-10**	2	38.9	38.1	38.3	39.9		
	4	34.6	34.4	UD	UD	UD	UD
	6	38.5	38.1	35.8	36.4		
	8	UD	UD	UD	UD	23.2	23.1
	10	25.8	25.8	UD	UD		
	12	UD	UD	UD	UD	UD	UD
	14	24	24.9	22.9	22.6		
	16	N/A	N/A	N/A	N/A	N/A	N/A
	18	N/A	N/A	N/A	N/A		
**BTV-17**	2	37.7	37	38.7	38.5		
	4	35.2	35.7	27.8	27.9	38.3	37.2
	6	31.6	31.7	31.8	31.7		
	8	24.1	24.6	36.1	37.5	35.5	35.9
	10	28.2	28.2	29.5	30		
	12	19.3	19.3	38.4	38	20.6	21.1
	14	21.7	21.5	22.5	25.6		
	16	UD	UD	23.3	20.8	23.7	21.2
	18	N/A	N/A	N/A	N/A		
**30C Temperature**		**Pool A**		**Pool B**		**Pool C**	
**Infection Group**	**DPI**	**ct1**	**ct2**	**ct1**	**ct2**	**ct1**	**ct2**
**BTV-10 & 17**	2	29.2	29.2	30	30		
	4	30.5	30.5	22.5	22.6	24.6	24.5
	6	20.3	20.7	24.6	25.3		
	8	24.2	19.8	23.5	22.6	20.8	20.5
	10	21.2	21.6	21.5	21.6		
	12	35.4	36.9	20.2	20.1	27.9	28.1
	14	22	21.5	22.6	22.6		
	16	N/A	N/A	N/A	N/A	N/A	N/A
	18	N/A	N/A	N/A	N/A		
**BTV-10**	2	UD	UD	UD	UD		
	4	UD	UD	UD	UD	33.3	33.3

	6	UD	UD	24.4	24.3		
	8	39	39.6	N/A	N/A	N/A	N/A
	10	N/A	N/A	N/A	N/A		
	12	N/A	N/A	N/A	N/A	N/A	N/A
	14	N/A	N/A	N/A	N/A		
	16	N/A	N/A	N/A	N/A	N/A	N/A
	18	N/A	N/A	N/A	N/A		
**BTV-17**	2	39.5	39.5	30.3	30.2		
	4	29.2	29.5	30	32.3	38.1	38.2
	6	33.8	25.7	35.1	34.8		
	8	UD	UD	21.9	22.5	35.7	35.9
	10	23.8	23.9	UD	UD		
	12	N/A	N/A	N/A	N/A	N/A	N/A
	14	N/A	N/A	N/A	N/A		
	16	N/A	N/A	N/A	N/A		
	18	N/A	N/A	N/A	N/A		
